# Rab25 as a tumour suppressor in colon carcinogenesis

**DOI:** 10.1038/sj.bjc.6605983

**Published:** 2010-11-09

**Authors:** J R Goldenring, K T Nam

**Affiliations:** 1 Nashville VA Medical Center and the Departments of Surgery and Cell and Developmental Biology, Epithelial Biology Center, Vanderbilt University School of Medicine, 10435G MRBIV, 2213 Garland Avenue, Nashville, TN 37232-2733, USA

**Keywords:** Rab proteins, colon cancer, vesicle trafficking, Rab11-FIP, integrin, Smad3

## Abstract

Recent investigations have increasingly focussed attention on the roles of intracellular vesicle trafficking in the regulation of epithelial polarity and transformation. Rab25, an epithelial-specific member of the Rab family of small GTPases, has been associated with several epithelial cancers. Whereas Rab25 overexpression is associated with ovarian cancer aggressive behaviour, Rab25 expression is decreased in human colon cancers independent of stage. Recent studies of mouse models of intestinal and colonic neoplasia have demonstrated that Rab25 deficiency markedly promotes the development of neoplasia. Some of these effects appear related to alterations in *β*1-integrin trafficking to the cell surface. These findings all suggest that Rab25 is a tumour suppressor for colonic neoplasia.

Transformation of cancer cells involves alterations in both cell shape and behaviour. Intrinsic to the pathophysiology of epithelial cancers is a loss of polarised epithelial monolayers and adoption of migratory cell behaviours that lead to locally invasive disease and metastasis. This transition in cell behaviour during carcinogenesis requires global changes in cell surface components that maintain the normal polarised phenotype including junctional proteins, integrins and plasma membrane receptors, pumps and channels. Over the past decade, research in a number of cell systems has revealed that directed trafficking of cell surface proteins is not a simple matter of protein synthesis and targeting. Rather, the phenotype of cell surface protein expression reflects a dynamic interplay of *de novo* delivery, internalisation, targeted degradation and recycling (Mellman and Nelson, 2008). At any given moment, the compendium of cell surface proteins present on normal or abnormal cells is determined by the net effects of all of these trafficking processes. It is therefore now increasingly clear that changes in critical trafficking pathways can induce alterations in cell surface proteins leading to alterations in polarity and susceptibility to further steps that may lead to transformation.

The importance of the loss of polarity in the generation of early neoplasia has received greater scrutiny over the past several years. The establishment of polarity with segregation of apical and basolateral membrane domains is perhaps the most obvious manifestation of polarity. The maintenance of the boundaries between these polarised membrane regions by components of the tight and adherens junctions is critical for control of the polarised epithelial barrier (Mellman and Nelson, 2008). Polarity also sets up critical functions of ligand movement between polarised surfaces (transcytosis) as well as polarised transport of ions and nutrients and polarised signalling through membrane receptors. The appropriate presentation of proteins and their density at the polarised surfaces markedly impacts the physiology of the epithelial monolayer. Loss of junctional components such as E-cadherin or p120 can lead to loss in epithelial polarity and eventual derangement of the epithelial monolayer, a precancerous event (Chen *et al*, 2003; Balzac *et al*, 2005; Lioni *et al*, 2007; Dohn *et al*, 2009; Smalley-Freed *et al*, 2010). As individual alterations of particular proteins may not be sufficient to alter general epithelial polarity, multiple derangements are likely required to induce the level of functional polarity loss necessary as an early step in carcinogenesis. Multiple hits are then likely to lead to transformation (loss of contact inhibition), invasion and metastasis.

The Rab proteins comprise the largest family of small GTPases, with over 60 mammalian gene products (Schwartz *et al*, 2007). Rab proteins associate with distinct membrane compartments within cells and are thought to represent the nidus for the assembly of multiprotein complexes that determine the specificity of vesicle trafficking pathways within cells (Somsel Rodman and Wandinger-Ness, 2000). In particular, the family of Rab11 GTPases, containing Rab11a, Rab11b and Rab25, is involved in the regulation of recycling of internalised membrane proteins (Ullrich *et al*, 1996; Casanova *et al*, 1999; Wang *et al*, 2000) as well as the movement of membrane proteins between polarised surfaces of epithelial cells (Jones *et al*, 2006). Although Rab11a and Rab11b are ubiquitously expressed, Rab25 is expressed only in epithelial cells (Goldenring *et al*, 1993). Rab25 contains an unusual WDTAGLE sequence in its GTP-binding domain. This sequence mimics the Q to L mutation seen in Ras and other Rab that confers a loss of GTPase activity (the dominant active phenotype). However, we have demonstrated previously that Rab25 retains an active GTPase activity (Casanova *et al*, 1999). Although expression of Rab25 is generally low, overexpression studies in MDCK cells indicated that Rab25 was a negative regulator of epithelial cell basolateral-to-apical transcytosis (Casanova *et al*, 1999; Wang *et al*, 2000). More recently, investigations by Lencer and colleagues (Tzaban *et al*, 2009) have demonstrated that Rab25 regulates apical-to-basolateral transcytosis in polarised MDCK cells. These findings suggest that Rab25 is an important regulator of polarised cell surface composition. Although most previous studies have focussed on investigation of model trafficking cargoes expressed in MDCK cells (e.g., polymeric IgA for basolateral-to-apical transcytosis and FcRN for apical-to-basolateral transcytosis of IgG), the endogenous cargoes for Rab25-dependent trafficking have remained generally obscure.

All of the Rab11 family members can interact with classes of Rab11-family interacting proteins (Rab11-FIPs) as well as the actin-based motors Myosin Va and Myosin Vb (Hales *et al*, 2001; Lapierre *et al*, 2001; Roland *et al*, 2009). *In vitro* data indicate that these proteins can bind to all three Rab11 family members, in the case of Rab11-FIPs, through a conserved amphipathic *α*-helix domain (Hales *et al*, 2001). Nevertheless, it is not at all clear whether there are real differences in affinity *in vivo*. A number of recent studies have implicated Rab11-family effector proteins in the regulation of the polarised cell phenotype. Phosphorylation of Rab11-FIP2 by Par1b/MARK2 appears to regulate the establishment of polarity in MDCK cells (Ducharme *et al*, 2006). Overexpression of Rab11-FIP2(S227A) inhibits the re-establishment of the polarised monolayer following disruption by calcium chelation. Loss of functional myosin Vb has also been identified recently as the key mutation in neonates with microvillous inclusion disease (MVID) (Erickson *et al*, 2008; Muller *et al*, 2008). The intestinal epithelia in MVID patients show a prominent loss of apical microvilli as well as apical membrane inclusions, which lead to devastating neonatal diarrhoea (Iancu *et al*, 2007). Recent studies have suggested that these losses in apical differentiation are associated with defects in polarity (Muller *et al*, 2008). Thus, alterations in membrane recycling system functions can lead to remarkable changes in polarised function. Nevertheless, it is important to note that in the case of MDCK cells expressing the Rab11-FIP2(S227A) mutation, once the epithelial polarity is established, the general functional characteristics of the resistance of the monolayer are essentially normal (Ducharme *et al*, 2006). Thus, single defects can be compensated for through other mechanisms. In addition, whereas defects in the intestinal epithelia of MVID patients are remarkable, kidney epithelia appear unaffected. Thus, different epithelial cells may have greater or lesser abilities to compensate for specific deficits.

A number of recent investigations have implicated Rab proteins in the process of carcinogenesis (recently reviewed in Chia and Tang, 2009). A number of studies have implicated the early endosomal Rab proteins Rab5 and Rab21 in the regulation of tumour cell behaviour (Pellinen *et al*, 2006, 2008; Torres *et al*, 2010). However, specific trafficking defects in recycling system function have not been studied extensively. It was therefore of interest in 2004 when Mills and colleagues (Cheng *et al*, 2004) noted that regions of chromosome 1 amplification associated with ovarian cancer were associated with marked increases in the expression of Rab25. In those investigations, Rab25 overexpression correlated with the aggressiveness of the cancer. The investigators also suggested that Rab25 was overexpressed in breast cancers. Nevertheless, in the past 2 years, it has become clear that Rab25 overexpression in breast cancer is likely less common and that indeed, at least in oestrogen receptor-deficient cancers, expression is actually decreased significantly (Cheng *et al*, 2006, 2010).

We have investigated the role of Rab25 in colon cancer patients (Nam *et al*, 2010). In two independent patient cohorts, gene microarray studies demonstrated that Rab25 expression was decreased in colon cancers independent of clinical and pathological staging. Importantly, Rab25 loss was associated with poorer survival. Interestingly, we did not observe any significant changes in the expression of Rab11a, Rab11b, any of the Rab11-FIP proteins or myosin Vb. These results suggested that loss of this epithelial-specific Rab protein was associated with an increased susceptibility to colon carcinogenesis.

To address the impact of loss of Rab25 on colon carcinogenesis, we developed a novel mouse model for genetic deletion of Rab25. Although we did not observe any effects of Rab25 loss in the knockout mouse strains themselves, when these mice were crossed onto mice with established susceptibility to developing intestinal neoplasms, we observed marked acceleration of tumourigenesis. In the case of the adenomatous polyposis coli model Apc^Min^ mice, breeding onto the Rab25 knockout background led to a four-fold increase in polyp number in the intestines and a two-fold increase in the tumour number in the colon (Nam *et al*, 2010). Interestingly, these polyps retained relatively well-differentiated morphologies of adenomatous polyps and did not show any increase in invasive properties. These results supported a concept that Rab25 promoted polyp initiation.

Although the Apc^Min^ mouse is a good model for polyposis (D’Abaco *et al*, 1996), it is less suitable to address colon carcinogenesis. We therefore also examined the influence of Rab25 loss on colon tumourigenesis in the Smad3 knockout mouse model (Zhu *et al*, 1998). Smad3 knockout mice develop colon tumours by 6 months of age. Heterozygous Smad3^+/−^ mice do not develop any pathology. We therefore were surprised to find that >80% of Smad3^+/−^; Rab25^−/−^ mice demonstrated large invasive colon tumours throughout the colon by 15 weeks of age. Interestingly, we could still demonstrate, although reduced, Smad3 expression in the tumours, and hence the effects appeared to be synergistic. Although it is presently not clear what mechanisms account for the remarkable increase in colon carcinogenesis in this model, it is important to note that the Smad3^+/−^; Rab25^−/−^ mice may represent a critical new model for colon carcinogenesis. Whereas Smad3^−/−^ mice show a marked overall deficit in weight gain throughout life, the Smad3^+/−^; Rab25^−/−^ mice grow similarly to wild-type mice. The highly invasive, and perhaps locally metastatic, character of the tumours and their rapid and highly penetrant phenotype makes this model perhaps the most adaptable model for the study of colon carcinogenesis in mice. The size of the tumours developed will likely be amenable to imaging and endoscopy approaches and could be of high utility in preclinical studies of novel colon cancer treatments (Deane *et al*, 2007).

What alterations in Rab25 knockout mice could be responsible for the increased susceptibility to intestinal and colon neoplasia when a second deficiency is added (either mutant Apc or reduction in Smad3)? Norman and colleagues (Caswell *et al*, 2007) have previously demonstrated that increases in Rab25 in ovarian cancer cells were associated with aberrations in the cell surface localisation of *β*1-integrin. We therefore evaluated the expression of *β*1-integrin in the Rab25-deficient mouse intestine. Whereas in wild-type mice *β*1-integrin was strongly expressed in the cells of the villus, we observed a decrement in basolateral *β*1-integrin and an increase in intracellular *β*1-integrin in the Rab25-deficient mice (Nam *et al*, 2010). Interestingly, we also observed the highest concentrations of Rab25 expression in the cells in the transition between the crypt and villus regions of the intestinal mucosa. This is the critical region for the upregulation of *β*1-integrin expression in the mucosa and is also the region implicated in the formation of adenomas in Apc^Min^ mice. Thus, a loss of proper *β*1-integrin trafficking to the basolateral membranes during maturation of cells from the crypt to the villus may provide the predisposing decrement that, with the addition of Apc loss, could lead to increased polyp formation. Interestingly, in the case of Apc^Min^ mice, we also observed that Rab25 heterozygote mice, which display an intermediate amount of Rab25 expression, showed an intermediate increase in polyp formation (Nam *et al*, 2010). Combined with the results in ovarian cancer cells, it appears that manipulation of Rab25 levels can lead to aberrations in the trafficking of *β*1-integrin and perhaps other critical regulators of cell adhesion (Figure 1). Nevertheless, although evidence does exists for the regulation of integrin trafficking in non-polarised cells by Rab11a (Mitchell *et al*, 2004), the exact mechanism that could account for the alterations of integrin trafficking remain unclear. Norman and colleagues (Caswell *et al*, 2007) have observed that under certain circumstances, some overlap of internalised *β*1-integrin and Rab25 can be observed; however, we have not been able to demonstrate colocalisation of *β*1-integrin with Rab25 in intestinal systems. Norman *et al* (Caswell *et al*, 2007) have also shown evidence for a possible direct interaction of Rab25 with *β*1-integrin. Nevertheless, the effects of Rab25 expression changes on integrins may also reflect *en passant* effects because of either alteration in the trafficking of another cargo (e.g., the EGF receptor) or perhaps by changing the effective concentration of direct effectors shared with Rab11a (e.g., Rab11-FIPs; Figure 1). Alternatively, the alterations seen with loss of Rab25 could reflect an alteration in the downstream effects mediated by Rab11-FIP1C/RCP. Although we did not observe any alterations in expression of Rab11-FIP1C in human colon cancers, recent investigations in breast cancer have implicated overexpression of Rab11-FIP1C in breast cancers (Zhang *et al*, 2009). In addition, these studies have suggested that Rab11-FIP1C can interact with and regulate activation of Ras (Figure 1). Similarly, in colon cancers, changes in Rab25 expression may lead to alterations in Ras through changes in competition between Rab25 and Ras for Rab11-FIP1C.

Thus, although it is clear that Rab25 can impact epithelial cell transformation, the exact mechanism remains elusive. It appears that the effects of Rab25 expression depend largely on context. Thus, either overexpression or loss of Rab25 expression can lead to transformation. It seems likely that these findings result from cell-specific roles for Rab25 in the trafficking of specific cargoes. Thus, if, as is documented in MDCK cells (Tzaban *et al*, 2009), Rab25 is involved in apical-to-basolateral transcytosis, either overexpression or loss of expression could lead to imbalances in the distribution of trafficking cargoes between the apical and basolateral membranes. Imbalances on either end of the spectrum could lead to loss in polarity that predisposes to transformation. Similarly, if trafficking outcomes depend on a competition between Rab25 and Rab11a for Rab11-FIPs (Figure 1), then overexpression or loss of Rab25 expression could have marked effects on the flow through membrane recycling systems of multiple cargoes, including signalling receptors such as the EGF receptor or TGF-*β* receptors. In considering these rather subtle effects, it is important to note that small defects in the efficiency of dynamic trafficking over an extended period of time may lead to progressive deficits, perhaps contributing to the age-related increase in cancer incidence.

## Conclusion

Loss of Rab25 in human colon cancers is associated with poorer patient prognosis. Although Rab25-deficient mice do not develop spontaneous colon tumours, breeding of Apc^Min^ mice onto the Rab25-deficient background induces a marked increase in tumours, and crossing of Smad3^+/−^ mice onto the Rab25 knockout background yields large invasive tumours. These results indicate that Rab25 is an important tumour suppressor in the colon. Given the results in both ovarian and breast cancer studies, it is likely that Rab25 influences the trafficking and recycling of a number of key regulators of polarity and signalling involved in transformation. Thus, either loss of Rab25 or overexpression of Rab25 may lead to cell transformation based on the cell-specific alteration in surface protein delivery.

## Figures and Tables

**Figure 1:**
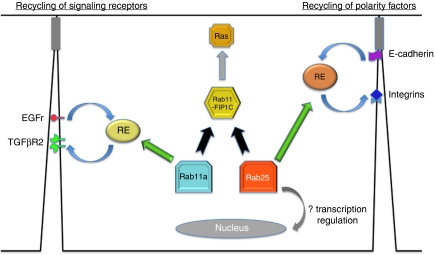
A scheme for regulation of transformation and cell polarity by Rab25. The figure shows the possible interactions of vesicle recycling through recycling endosomes (RE) of either signalling receptors (on the left) or polarity-related proteins (on the right). Loss of Rab25 in colon cells could lead to either direct dysregulation of these pathways or imbalances in parallel trafficking pathways regulated by Rab11a or by alteration of influences on downstream shared effectors such as Rab11-FIP1C (also known as RCP). Effects of Rab25 on transcription remain unknown, although there is no evidence that Rab25 ever enters the nucleus.
